# The surgical strategy of Purpura fulminans triggered by pyothorax associated with lung cancer

**DOI:** 10.1080/23320885.2019.1704290

**Published:** 2019-12-23

**Authors:** Yoshitaka Matsuura, Katsuya Kawai

**Affiliations:** a Department of Plastic and Reconstructive Surgery, Graduate School of Medicine, Kyoto University, Kyoto, Japan; b Department of Plastic and Reconstructive Surgery, Nagahama Red Cross Hospital, Shiga, Japan

**Keywords:** Purpura fulminans, pyothorax, combination therapy, walk

## Abstract

Purpura fulminans is a rare disease that usually causes sepsis and is accompanied by disseminated intravascular coagulation and symmetric gangrene of distal extremities. We had to consider the most appropriate surgery approach. The most important point was attempting to rescue the patient’s ability to walk under his own power.

## Introduction

Purpura fulminans (PF) is a rare disease usually associated with disseminated intravascular coagulation (DIC) and accompanied by symmetric gangrene of the distal extremities. The etiology of the disease remains unclear. There are three kinds of PF: congenital, acute infectious and idiopathic PF [1]. Congenital PF is an inherited derangement of the coagulation cascade, such as in cases of the absence of protein C or protein S. Acute infectious PF is usually accompanied by sepsis. The diagnosis of idiopathic PF is made by exclusion and usually presents with less severe features than other types of PF [1].

Acute infectious PF is caused by sepsis, carrying a mortality of about 40% [2]. For this reason, it has been considered a kind of severe sepsis. In the present case report, a 41-year-old man suffered sepsis shock, adrenal failure and PF triggered by pyothorax associated with lung cancer. All four of his limbs developed necrosis. We describe the surgical strategy for treating PF with pyothorax and include the treatment results.

## Case report

**Figure 1.**
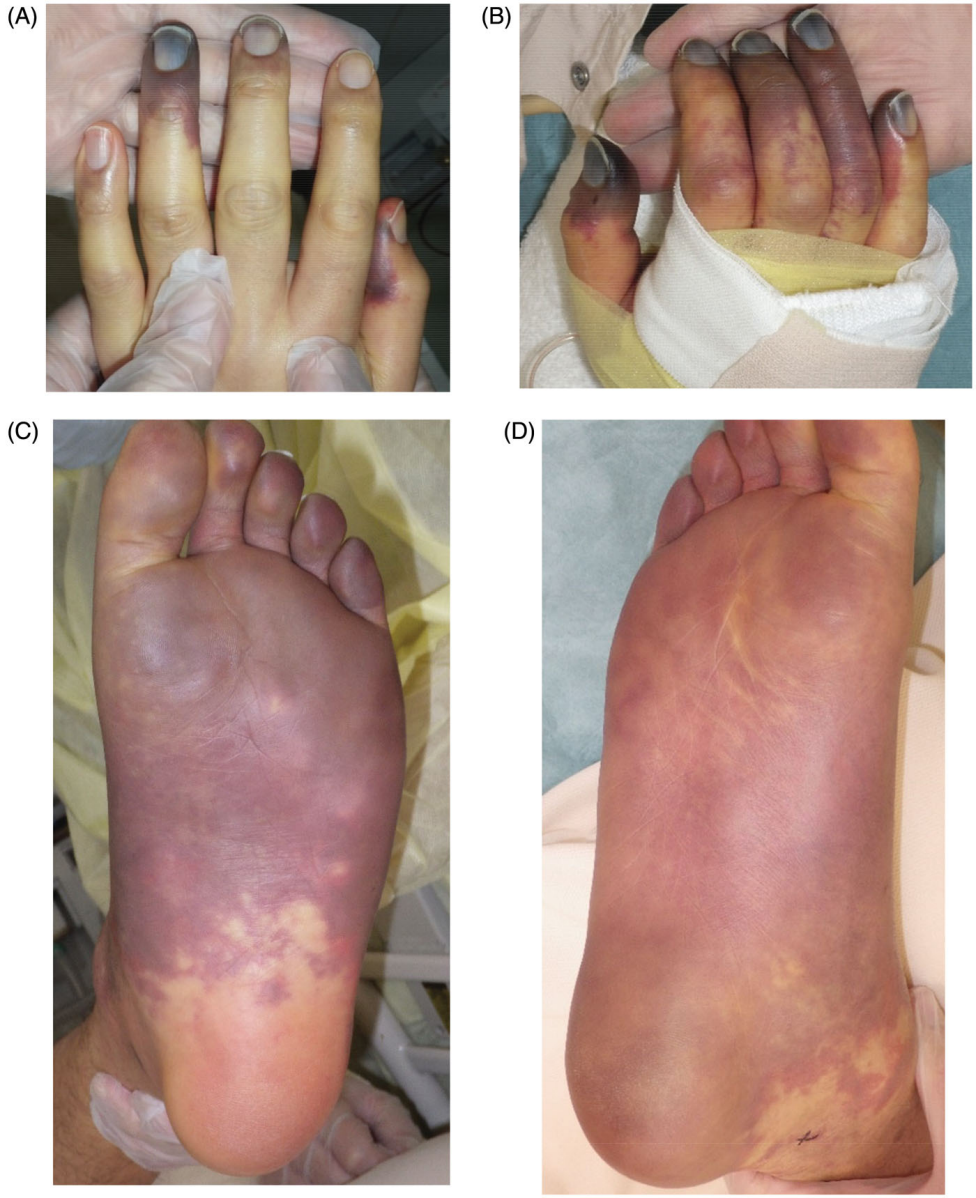
The conditions at day 5 of hospitalization are shown. The purpura had expanded to both hands and feet. (A) Left hand, (B) right hand, (C) left foot, (D) right foot.

A 41-year-old man had pyothorax after surgery and chemotherapy for lung cancer. He developed sepsic shock and DIC 13 months after undergoing respiratory surgery to excise lung cancer. He was admitted to the intensive-care unit and required dialysis for renal failure. His condition worsened as he developed accompanying PF that affected both hands and feet (Figure 1). *Methicillin-Sensitive Staphylococcus Aureus* detected in the pyothorax was considered the main pathogen responsible for sepsis.

**Figure 2.**
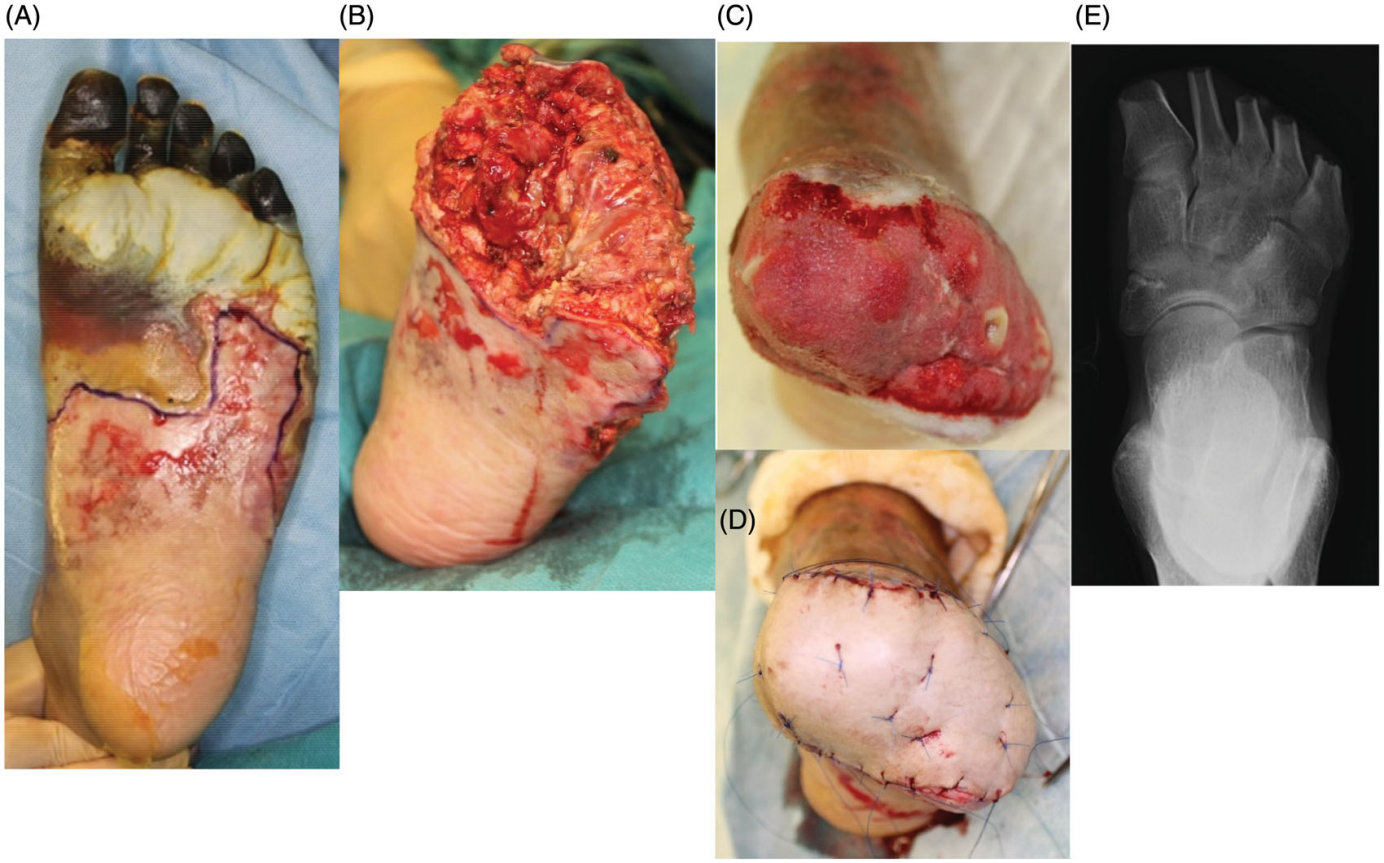
The left foot was amputated at the level of the metatarsal bone. All necrotic tissue was excised. Negative pressure wound therapy (NPWT) was applied to any wounds associated with exposed bone to speed up the healing process and cover the bone with granulation tissue as a wound bed preparation. After three weeks, there was good granulation, with the bone almost buried. Finally, a partial-thickness skin graft was applied to the wounds. (A) Before amputation, (B) after amputation, (C) condition at three weeks after NPWT, (D) skin graft on wound, (E) X-ray findings after surgery.

Part of the purple-colored skin changed to black, indicating total necrosis. This brought up the issue of how to proceed with surgical treatment for multiple-leg necrosis considering the patient’s pyothorax, which carried a risk of worsening the general condition. Although general anesthesia was possible, we planned to perform the surgery with local and spinal anesthesia in order to minimize the risk of complications, such as pneumonia, after the surgery. The ultimate goal was for the patient to be able to walk again under his own power.

First, we decided to save as much of the left leg as possible, as the necrosis did not involve the heel (Figure 2(A)). The left foot was therefore amputated at the level of the metatarsal bone, and all necrotic tissue was excised (Figure 2(B,E)). The exposed bone associated with the stump presented a problem in connection with the next surgery, skin graft. As a result, negative pressure wound therapy (NPWT) was applied to the wound exposed bone in order to speed up the healing process and cover the bone with granulation tissue. The effectiveness of this therapy for wounds with exposed bone in the lower extremities has been previously described [3]. The device we used was an ACTIV.A.C^®^ (K.C.I, San Antonio, USA), and the pressure was adjusted 50 mmHg. After three weeks, the wound improved and exposed bone was now almost completely covered with granulation tissue. The wound bed preparation before performing skin grafting was thus considered to be sufficient (Figure 2(C)). Finally, a partial-thickness skin graft (20/1000 inch) was used to cover the wound (Figure 2(D)). All of the engrafted skin survived.

**Figure 3.**
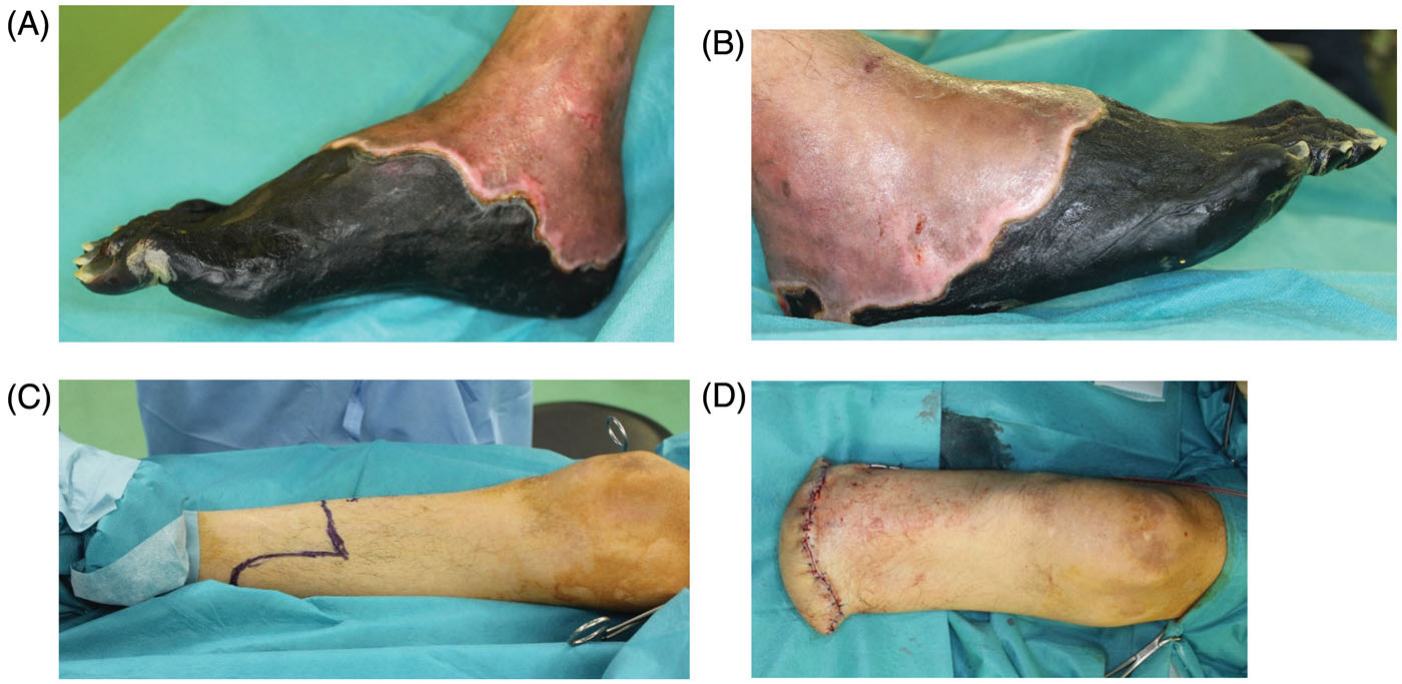
The necrotic tissue expanded from the fingers to the right heel. Amputation was performed at the level of the lower leg, and the wound was closed primarily. (A)(B) The condition before amputation, (C) amputation design, (D) condition after suturing.

Unfortunately, regarding the right foot, the necrotic tissue had expanded from the toes of the foot to the heel in the right foot (Figure 3(A,B)). Below-the-knee amputation was therefore required in order to excise all of the necrotic tissue, including the heel. The wound was able to be closed primarily (Figure 3(C,D)).

**Figure 4.**
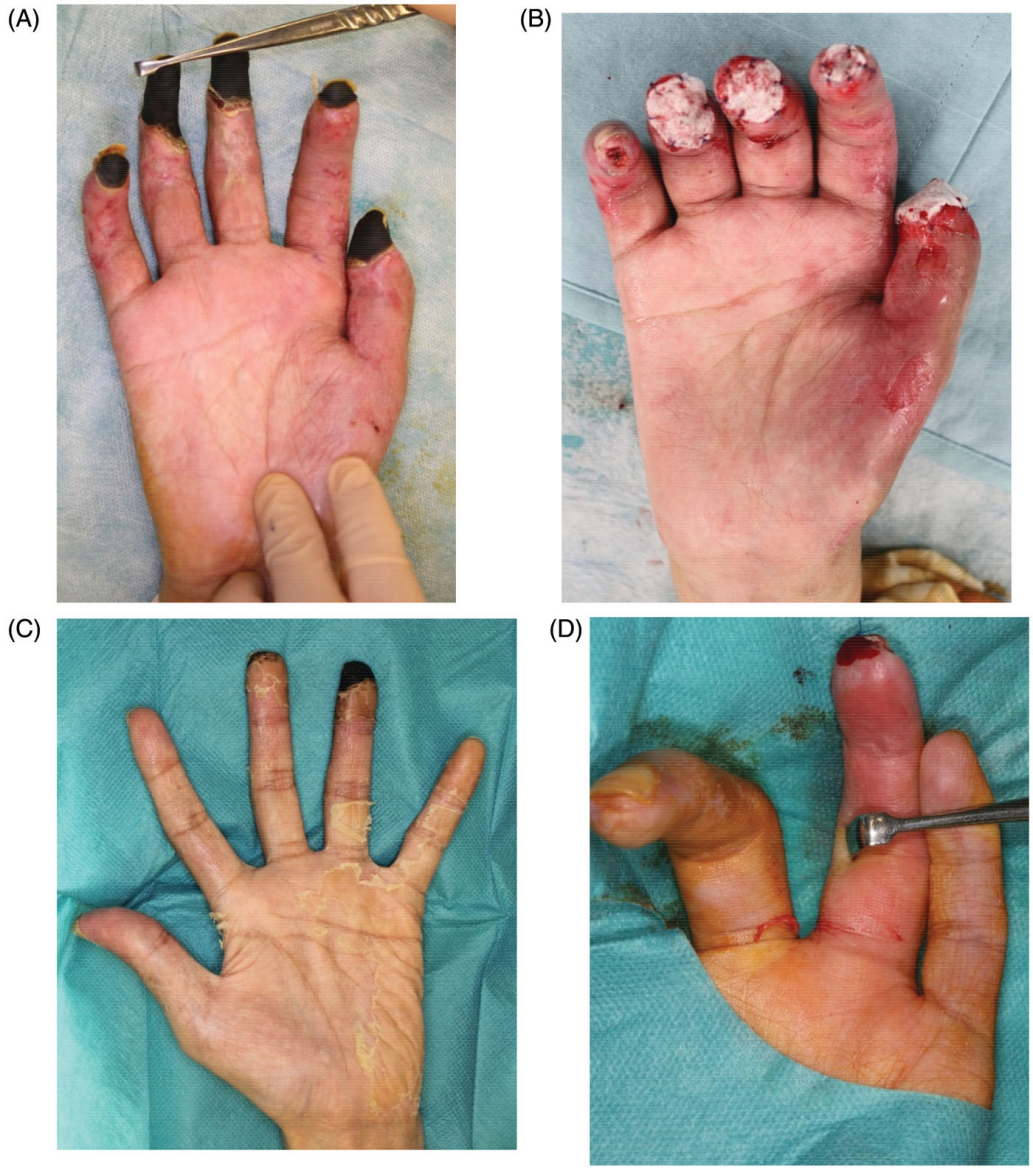
All five fingers on the right hand and the ring finger on the left hand showed necrosis. All necrotic tissues, including the bone, were excised. Artificial dermis was used to cover the wounds of the right hand after amputation. (A) The right hand before amputation, (B) right hand with artificial dermis, (C) left hand before amputation, (D) left ring finger after amputation.

**Figure 5.**
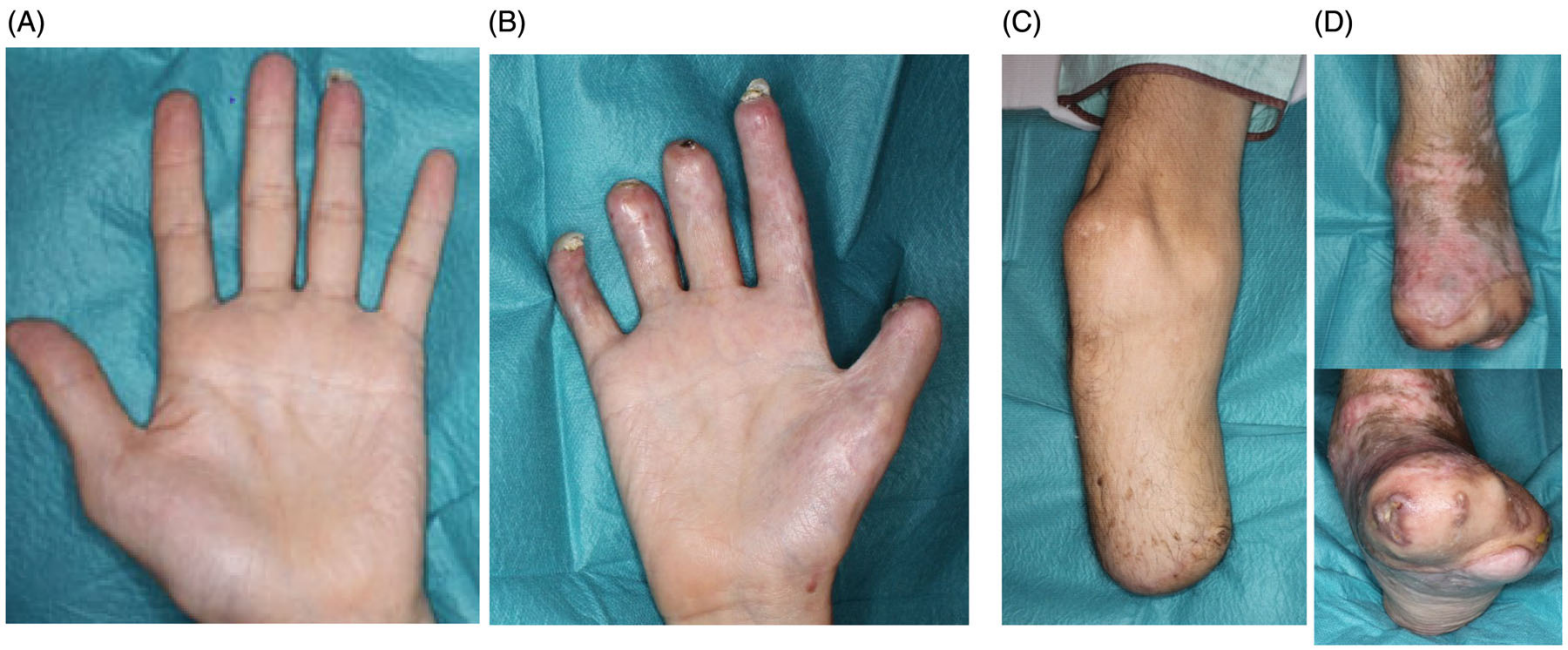
The condition at four months after the last surgery. All wounds had already healed completely. (A) The left hand, (B) right hand, (C) right foot, (D) left foot.

**Figure 6.**
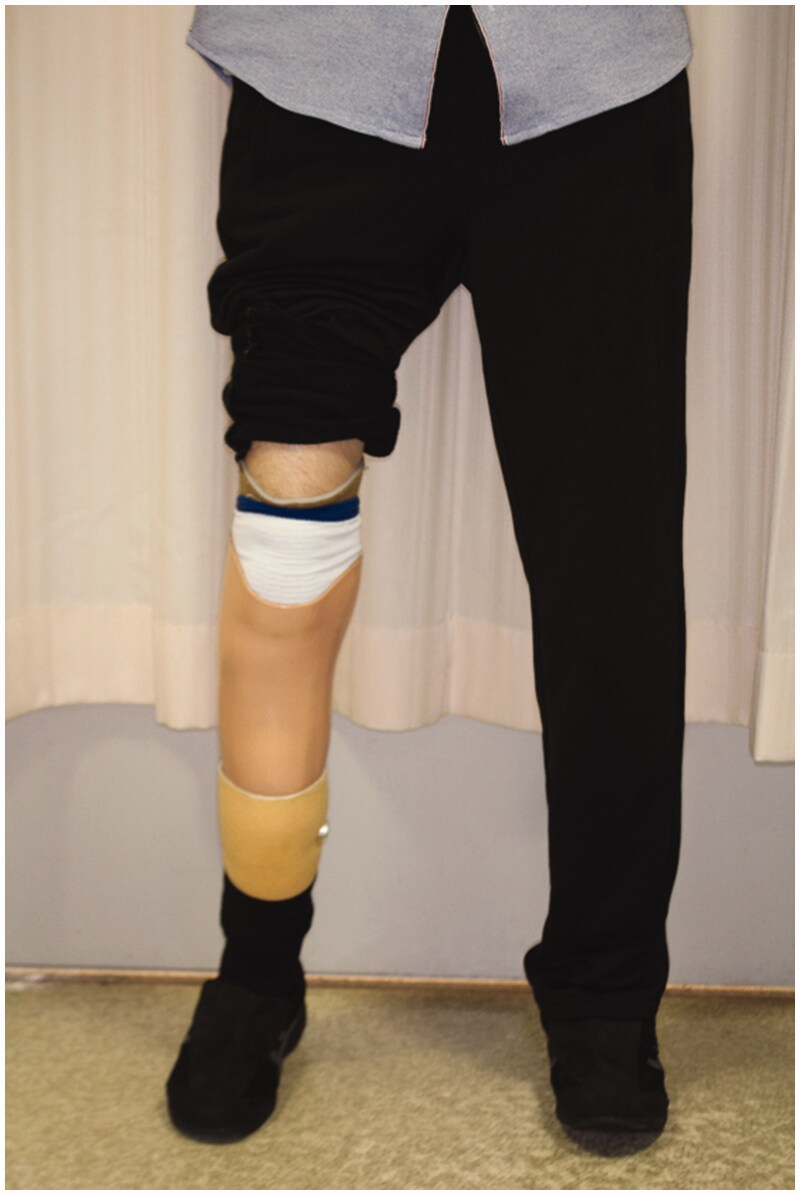
Patient’s condition while standing. The patient was able to walk by himself using a prosthesis for his right foot. He does not require a prosthesis for the left foot.

The necrotic parts of both hands were limited to the fingers. All five fingers on the right hand and the ring finger on the left hand showed necrosis. All necrotic tissues were excised or amputated with the bone (Figure 4(A,B,D)). PELNAC^®^ (GUNZE, Tokyo, Japan), which is a kind of artificial dermis, was used to cover the wounds at the right hand after amputation (Figure 4(C)).The condition at four months after the last surgery shows (Figure 5). All wounds on the both hands and feet had already healed completely. Rehabilitation restored his ability to walk, and he was ultimately able to walk by himself after fitting with an artificial prosthesis at the amputated right limb (Figure 6).

## Discussion

PF causes symmetric gangrene of distal extremities without proximal vascular occlusion. Although the mechanism remains unclear, Nolan noted that PF was associated with a low level of protein C and the absence of a spleen [4]. Protein C is a vitamin K-dependent glycoprotein with anticoagulant properties as well as anti-inflammatory properties, which may contribute to improved survival [5]. The spleen has important immune functions, such as phagocytosis and bacterial filtration. Therefore, patients after splenectomy are recommended to undergo vaccination against *Streptococcus pneumoniae* [6]. However, the present patient did not have low levels of protein C, nor had he undergone splenectomy.

Acute infectious PF is a rapidly progressive infectious disease involving a large amount loss of tissue and DIC [7]. Symmetric gangrene of the distal extremities occurred and it worsened over time due to the onset of total necrosis during the course of the disease. Childers reported a mortality rate of 43% in their retrospective review of 28 cases [8]. Even with life-saving intensive-care treatment, survivors must still have necrotic tissues excised. Warner described a high incidence rate for amputation (90%) [2]. Patients with amputated legs clearly have a reduced quality of life compared to those without amputated legs. However, the most important point is whether or not they can regain the ability to walk by themselves. Therefore, plastic surgeons should set the ultimate goal in such patients as ‘walking’ when performing surgical treatment in cases of acute infectious PF.

In the present case, we planned the surgical operation while taking into account his respiratory condition, particularly the presence of pyothorax. The patient was in a fairly poor condition after his previous respiratory surgery. We wanted to avoid administering systematic anesthesia, so our surgery plan involved the combination of local, spinal and/or transmission anesthesia. We also applied artificial dermis to the fingers of the hands after removing the necrotic tissue, performed below-the-knee amputation at the right leg, delivered vacuum-assisted therapy and placed a skin graft at the left leg.

Vacuum-assisted therapy can help promote granulation rapidly and decrease the surface wound area. The vacuum pressure is adjustable from 50 to 125 mmHg. We selected 50 mmHg in the present patient because we worried that the skin perfusion might be lower than usual due to potential skin damage from acute infectious PF. DeFranzo reported it was effective for promoting granulation on wounds with exposed bone in the lower-extremities according to the findings of his 75-patient series [3]. The granulation with this system was very good, and the exposed bone at the wound was almost completely buried by granulation. Furthermore, the wound bed preparation was so good before the start of skin grafting that the take after transplantation could thus be successfully completed. Artificial dermis has also been shown to be effective for exposed bone as well. PELNAC^®^ is a kind of artificial dermis composed of pig atelocollagen without the addition of chondroitin sulfate [9–11]. The treatment of wounds with bone exposure using artificial dermis has been reported [12–14]. Extensive wounds require skin grafts for closure after granulation has been promoted with artificial dermis. In the present case, however, we were able to completely close the small area at the tip of the finger, so a skin graft was not needed. We believe that both vacuumed-assisted therapy and artificial dermis are effective for managing wounds with exposed bone.

## Conclusion

We experienced a severe case of acute infectious PF triggered by pyothorax. Our combination therapy approach involving below-the-knee amputation, metatarsotomy, vacuumed-assisted therapy and the use of artificial dermis and skin grafts was effective. The patient’s condition improved, and he was ultimately able to walk by himself.
